# Environmental factors influencing mucilage accumulation of the endangered *Brasenia schreberi* in China

**DOI:** 10.1038/s41598-018-36448-3

**Published:** 2018-12-18

**Authors:** Chun Xie, Jiafeng Li, Fan Pan, Junjie Fu, Wenzong Zhou, Shan Lu, Pengfu Li, Changfang Zhou

**Affiliations:** 1 School of Life Sciences, Nanjing University, Nanjing, 210023 P.R. China; 2 Eco-environmental Protection Research Institute, Shanghai Academy of Agricultural Sciences, Shanghai, 201403 P.R. China

## Abstract

*Brasenia schreberi* J. F. Gmel. (Cabombaceae), a perennial freshwater macrophyte characterized by a thick mucilage on all underwater organs and especially young buds, has been widely cultivated as an aquatic vegetable in China for many years but is now listed as an endangered species due to anthropogenic impacts and habitat loss. Recent studies have demonstrated that different *B*. *schreberi* populations in China have low levels of genetic diversity but significantly different mucilage contents (MucC). Considering the importance of mucilage on both economic and ecological aspects, we examined mucilage-environment relationships in three *B*. *schreberi* cultivation sites. The results indicated that water permanganate index (COD_Mn_), total N (TN_w_), electrical conductivity (EC_w_), dissolved oxygen (DO_w_), sediment organic carbon (SOC) and total N (TN_s_) were significant factors, which explained 82.2% of the variation in mucilage accumulation. The MucC and mucilage thickness (MucT) as well as single bud weight (SBW) of *B*. *schreberi* showed negative relationships with COD_Mn_, TN_w_ and EC_w_ but positive relationships with SOC and TN_s_. Besides, high temperature may have a negative impact on mucilage accumulation of the species. Our study demonstrated that the mucilage accumulation of *B*. *schreberi* required good water quality and nutrient-enriched sediments, suggesting that habitat conservation, especially the quality of water, is important for maintaining *B*. *schreberi* populations.

## Introduction

Wetlands are one of the most important and biologically diverse ecosystems on earth, providing numerous essential ecosystem services and representing significant economic values^1,2^. Aquatic macrophytes are not only primary producers within wetlands, with their growth being affected by multiple environmental factors of the habitats, they are also important indicators of the health status of ecosystems^3^. The composition and distribution of the aquatic macrophyte community can be influenced by hydrology, substrate type, watershed, land use situation, water and sediment chemistry, etc^4–7^. Organic matter and nutrient levels in sediments were found to have important effects on macrophyte growth^4,5^. The turbidity (Turb_w_), transparency, electrical conductivity (EC_w_) and nutrient levels in water were also found to play a major role in macrophyte abundance^8–10^. Furthermore, geographic isolation had a large influence on the genetic differentiation of aquatic macrophytes^11^. With the wide recognition of global warming in recent decades, some reports also suggest that climate warming may influence the growth and distribution of macrophytes^12–14^.

*Brasenia schreberi* J.F. Gmel. (Cabombaceae), also known as watershield, is a perennial floating-leaved aquatic macrophyte. It has important value in taxonomic studies^15^ and is widely but sporadically distributed in freshwater ponds in tropical and temperate regions of East Asia, the West Indies, Australia, North and Central America, etc^16,17^. *B*. *schreberi* is a good indicator of the environments as it favours wetlands with clean water^18^. Due to extremely low seed germination rates, clonal reproduction by winter buds (or turions) is the main reproductive strategy in this species^19^. *B*. *schreberi* has been extensively cultivated in China for many years, and the young leaves and buds are an important vegetable in the diet^20,21^. However, *B*. *schreberi* has been recently listed as a critically endangered species in several countries of East Asia, as the wild populations of *B*. *schreberi* have decreased sharply due to habitat fragmentation and loss, human disturbances and vegetative propagation, etc^17,22^. In China, conservation zones for the germplasm resources of *B*. *schreberi* have been established^18^, but the trend of population declines still need to be reversed. Studies on *B*. *schreberi* have revealed low levels of genetic diversity and similar genetic structure for different cultivation populations in China^22,23^, as well as low gene flow rates among populations in Korea^17,24^, which may result in weak resistance to environmental change^22^. Studies on cultivation areas of *B*. *schreberi* in China have also reported relatively low species diversity at the community level^25^.

As an important aquatic vegetable, *B*. *schreberi* is characterized with its mucilage, which covers the submerged organs of the plant, including young stems, leaves and buds, and becomes less abundant as leaves age. People normally use mucilage content (MucC) to compare quality of the vegetable from different cultivation areas^26^. The mucilage itself is a mixture of acidic polysaccharides, hot water-soluble polysaccharides, proteins, polyphenol and trace elements^27,28^ and because of its hygienical functions, it is used for health purposes in food and medicinal industries in China^21^. Besides, mucilage exhibits excellent lubricating behaviour and may be used in the mechanical industry in the future^21,29^. Meanwhile, the mucilage produced by *B*. *schreberi* has its ecological meaning, it can reduce herbivory on leaves of the plant during growing season^30^. As an important photosynthetic product, mucilage can also reflect the growth status of *B*. *schreberi* to a certain extent. Based on the low levels of genetic diversity among these populations, we suppose that the discrepancy on mucilage accumulation of *B*. *schreberi* from different cultivation areas may be related to environmental conditions. However, little attention has been paid to the relationships between the environmental factors (for example, climate, water, and sediment) and *B*. *schreberi* growth. In addition, with habitat degradation and fragmentation in wetlands, studies that comprehensively consider the effects of multiple environmental factors on the growth of *B*. *schreberi* are particularly important, as these studies can provide critical information for the conservation of the endangered species.

In this study, we hypothesized that (1) good water quality and nutrient availability in both water and sediments support the mucilage accumulation in *B*. *schreberi* and (2) air temperature in growth seasons also affects the quality. To test these hypotheses, we investigated the environmental factors and analysed the watershield quality in Suzhou (SZ), Hangzhou (HZ) and Lichuan (LC) in China. These areas are currently the three largest cultivation centres in the country, as SZ and HZ are historical centres with cultivation records of more than 1500 years, but now have less than 1 km^2^ in each site, and LC is a new centre with a cultivation history of approximately 30 years but has more than 20 km^2^ at present. Considering that the water quality, air temperature and properties of the watershield may fluctuate considerably during growing seasons, related parameters were repeatedly analysed in spring, summer and autumn, whereas sediments were relatively stable and only analysed in spring.

## Results

### Plant properties

The seasonal changes in watershield quality are illustrated in Table 1. Mucilage content (MucC) is the percentage of mucilage in buds, which is the key component that measuring the quality of watershield as vegetable. MucC in LC was the highest among the three sites. MucC values in SZ and LC were relatively low in spring but increased in summer and autumn, whereas HZ had the lowest MucC in summer. Mucilage thickness (MucT) is the thickness of mucilage adhering to petiole, which is negatively correlated with herbivore damage. The MucT in LC in summer and autumn increased dramatically compared to the level in spring, so it was significantly higher than that in SZ and HZ in the same season. A slight increase in MucT in SZ was also revealed in autumn, whereas the MucT in HZ decreased throughout the growing seasons. Single bud weight (SBW) is the average weight of each bud, which not only influences the total mucilage production and also indicates the growth condition of the plant. Although relatively smaller in spring, a significant increase in SBW was found in LC in summer and autumn. The SBW values of SZ and HZ were both lowest in summer. Rolled-leaf length (RLL) is used as another index of the grow condition of the plant, which is positively correlated with the leaf area when expand, and also contributes to the mucilage accumulation indirectly. There were no general trends on RLL along sites or seasons. Across the growing seasons, SZ and HZ had relatively greater variation in RLL than LC. Except for the effect of season on RLL which was not significant, the effects of season, site and their interactions on all the remaining plant properties were significant.

**Table 1:** Differences in mucilage and relative plant properties among sampling sites across growing seasons. Significance of the variance was analysed by two-way ANOVA.

Plant parameters	Sites	Mean ± SD			Significance			
		Spring	Summer	Autumn	Source	df	*F*-value	*P*-value
Mucilage content (MucC, %)	SZ	43.0 ± 5.7^Bb^	54.4 ± 5.0^Ab^	58.8 ± 4.5^Ab^	Site	2	21.594	<0.001
	HZ	60.2 ± 4.8^Aa^	47.7 ± 9.7^Bb^	59.7 ± 4.6^Ab^	Season	2	4.956	0.035
	LC	64.2 ± 9.7^Aa^	70.8 ± 3.7^Aa^	71.2 ± 3.3^Aa^	Site × Season	4	5.410	0.017
Mucilage thickness (MucT, mm)	SZ	1.12 ± 0.18^Bb^	1.02 ± 0.25^Bb^	1.50 ± 0.32^Ab^	Site	2	158.353	<0.001
	HZ	1.51 ± 0.27^Aa^	0.93 ± 0.16^Bb^	0.75 ± 0.22 ^Cc^	Season	2	11.052	0.004
	LC	1.30 ± 0.38^Cab^	2.03 ± 0.36^Ba^	2.38 ± 0.37^Aa^	Site × Season	4	63.849	<0.001
Single bud weight (SBW, g)	SZ	1.83 ± 0.35^Aa^	1.45 ± 0.27^Bb^	1.81 ± 0.20^Ab^	Site	2	82.418	<0.001
	HZ	2.19 ± 0.50^Aa^	1.25 ± 0.23^Bb^	1.73 ± 0.25^Ab^	Season	2	7.185	0.014
	LC	1.63 ± 0.35^Bb^	3.61 ± 0.47^Aa^	3.25 ± 0.55^Aa^	Site × Season	4	41.343	< 0.001
Rolled-leaf length (RLL, mm)	SZ	46.89 ± 7.32^Cb^	51.75 ± 8.26^Ba^	57.79 ± 5.75^Aa^	Site	2	7.637	0.012
	HZ	54.68 ± 6.84^Aa^	43.85 ± 5.60^Bb^	44.48 ± 5.22^Bc^	Season	2	0.057	0.945
	LC	49.97 ± 6.73^Bb^	55.25 ± 5.04^Aa^	50.12 ± 4.86^Bb^	Site × Season	4	16.152	< 0.001

SZ, Suzhou; HZ, Hangzhou; LC, Lichuan. Data are listed as the mean ± standard deviation (SD). For MucC and SBW, *n* = 10; for MucT and RLL, *n* = 30. Different upper-case letters indicate statistically significant differences between seasons at the level *P* < 0.05, and different lower-case letters indicate statistically significant differences between sites at the level *P* < 0.05.

### Water parameters

The water parameters exhibited dramatic differences among the three sampling sites, and large fluctuations were also revealed from spring to autumn at the same site (Table 2). The water permanganate index (COD_Mn_) values of the three sites were similar in spring. Pronounced increases in COD_Mn_ were found in HZ and SZ from spring to autumn, but relatively small variations were found in LC, which had the lowest value in summer. The concentrations of total nitrogen (TN_w_) and total phosphorus (TP_w_) in the water for all sites generally dropped from spring to autumn, except for the TP_w_ of HZ, which was highest in summer. The values of water pH (pH_w_) for all sites were in the range of 7–8, with small but statistically significant differences between sites in the same season or between seasons in the same site. The water oxidation-reduction potential (ORP_w_) changed dramatically among different seasons in HZ and LC and was even below zero in HZ in summer. The ORP_w_ in SZ was relatively stable. The water dissolved oxygen (DO_w_) value in HZ was generally lower than that in SZ and LC, but extremely low values were found in both HZ in summer and LC in autumn. The Turb_w_ was generally at low levels for all three sites during the growing seasons, except the levels of LC in autumn, which were doubled compared with the data in earlier seasons. The EC_w_ value in SZ and HZ was considerably higher than that of LC. Meanwhile, the EC_w_ values in SZ and HZ changed significantly between seasons but were relatively stable in LC. Nevertheless, statistical analyses using two-way ANOVA revealed that not only season or site but also their interactions exerted significant effects on all water parameters, except their interactions on TN_w_.

**Table 2:** Differences in water parameters among sampling sites across growing seasons. Significance of the variance was analysed by two-way ANOVA.

Water parameters	Sites	Mean ± SD			Significance			
		Spring	Summer	Autumn	Source	df	*F*-value	*P*-value
Permanganate index (COD_Mn_, mg/L)	SZ	7.94 ± 0.09^Cb^	9.18 ± 0.42^Bb^	12.29 ± 1.28^Ab^	Site	2	260.717	<0.001
	HZ	8.42 ± 0.10^Ca^	13.91 ± 0.95^Ba^	17.61 ± 1.51^Aa^	Season	2	88.812	<0.001
	LC	7.38 ± 1.08^Aab^	4.96 ± 0.20^Bc^	6.16 ± 0.61^Ac^	Site × Season	4	50.493	<0.001
Total N (TN_w_, mg/L)	SZ	1.23 ± 0.10^Ac^	0.96 ± 0.19^Bb^	0.66 ± 0.11^Cb^	Site	2	31.168	<0.001
	HZ	1.78 ± 0.07^Aa^	1.52 ± 0.38^Aa^	1.46 ± 0.31^Aa^	Season	2	19.397	<0.001
	LC	1.42 ± 0.18^Ab^	0.84 ± 0.21^Bb^	0.62 ± 0.44^Bb^	Site × Season	4	1.427	0.245
Total P (TP_w_, μg/L)	SZ	37.8 ± 12.6^Ab^	32.8 ± 10.5^Ab^	12.6 ± 5.1^Bb^	Site	2	37.709	<0.001
	HZ	47.9 ± 7.6^Ab^	65.5 ± 21.1^Aa^	55.8 ± 16.1^Aa^	Season	2	7.888	0.001
	LC	109.2 ± 21.2^Aa^	65.5 ± 13.8^Ba^	58.0 ± 23.3^Ba^	Site × Season	4	6.311	0.001
pH (pH_w_)	SZ	7.79 ± 0.06^Aa^	7.46 ± 0.06^Bb^	7.39 ± 0.07^Cb^	Site	2	8.975	<0.001
	HZ	7.54 ± 0.10^Ab^	7.11 ± 0.20^Bc^	7.89 ± 0.58^Aa^	Season	2	6.609	0.002
	LC	7.01 ± 0.11 ^Cc^	7.64 ± 0.04^Aa^	7.42 ± 0.18^Bb^	Site × Season	4	42.744	<0.001
Oxidation-reduction potential (ORP_w_, mV)	SZ	185.1 ± 32.6^Ab^	203.1 ± 29.9^Ab^	201.2 ± 21.9^Ab^	Site	2	86.332	<0.001
	HZ	263.6 ± 19.4^Aa^	−111.2 ± 32.0 ^Cc^	228.9 ± 23.2^Ba^	Season	2	139.080	<0.001
	LC	170.7 ± 4.8^Bb^	258.8 ± 29.0^Aa^	1.3 ± 30.2 ^Cc^	Site × Season	4	590.381	<0.001
Dissolved oxygen (DO_w_, mg/L)	SZ	6.72 ± 0.71^Ba^	11.50 ± 1.90^Aa^	10.28 ± 1.46^Aa^	Site	2	437.365	<0.001
	HZ	4.26 ± 1.10^Ac^	0.59 ± 0.48 ^Cc^	3.38 ± 1.14^Bb^	Season	2	37.288	<0.001
	LC	5.92 ± 0.27^Bb^	8.09 ± 1.43^Ab^	0.49 ± 0.22 ^Cc^	Site × Season	4	134.325	<0.001
Turbidity (Turb_w_, NTU)	SZ	2.85 ± 2.06^ABa^	3.89 ± 1.65^Aa^	1.41 ± 0.30^Bc^	Site	2	13.230	<0.001
	HZ	2.61 ± 1.38^Aa^	3.06 ± 0.70^Aa^	2.30 ± 1.10^Ab^	Season	2	3.732	0.027
	LC	2.52 ± 0.41^Ba^	3.05 ± 0.77^Ba^	6.14 ± 1.92^Aa^	Site × Season	4	22.759	<0.001
Electrical conductivity (EC_w_, μS/cm)	SZ	142.2 ± 3.9^Bb^	135.0 ± 4.9^Cb^	226.3 ± 17.2^Aa^	Site	2	4406.897	<0.001
	HZ	244.2 ± 3.0^Aa^	239.9 ± 12.4^Aa^	82.5 ± 11.1^Bb^	Season	2	124.046	<0.001
	LC	33.1 ± 2.2^Bc^	35.2 ± 1.9^Ac^	33.1 ± 4.6^ABc^	Site × Season	4	1087.961	<0.001

SZ, Suzhou; HZ, Hangzhou; LC, Lichuan. Data are listed as the mean ± standard deviation (SD). For COD_Mn_, TN_w_ and TP_w_, *n* = 5; for pH_w_, ORP_w_, DO_w_, Turb_w_ and EC_w_, *n* = 15. Different upper-case letters indicate statistically significant differences between seasons at the level *P* < 0.05, and different lower-case letters indicate statistically significant differences between sites at the level *P* < 0.05.

### Sediment parameters

The sediment in SZ contained significantly lower nutrients compared to that in HZ and LC (Table 3). The values of sediment organic carbon (SOC) and sediment nitrogen (represented as total nitrogen (TN_s_) and available nitrogen (AN_s_)) in HZ and LC were 5 times those in SZ (*P* < 0.05). HZ also had the highest level of phosphorus (as total phosphorus (TP_s_) and available phosphorus (AP_s_)), followed by LC at approximately half the level of HZ (*P* < 0.05). SZ had the lowest level of phosphorus, and especially the AP_s_ in SZ, which was only 1/6 the level in HZ (*P* < 0.05). For potassium, different trends between total potassium (TK_s_) and available potassium (AK_s_) were found; HZ was still ranked highest in AK_s_, followed by LC. However, for TK_s_, the values from high to low were LC > HZ > SZ (*P* < 0.05). The sediments in SZ and LC were slightly acidic with sediment pH (pH_s_) values of approximately 5, whereas those in HZ were neutral with pH_s_ values of approximately 7 (*P* < 0.05).

**Table 3:** Differences in sediment parameters among sampling sites. Significance of the variance was analysed by one-way ANOVA.

Sediment parameters	Sites		
	Suzhou (SZ)	Hangzhou (HZ)	Lichuan (LC)
Sediment organic carbon (SOC, g/kg)	8.2 ± 3.7^b^	44.7 ± 11.3^a^	47.6 ± 2.2^a^
Total N (TN_s_, g/kg)	0.72 ± 0.22^b^	3.50 ± 0.97^a^	3.45 ± 0.37^a^
Total P (TP_s_, g/kg)	0.30 ± 0.02^c^	0.76 ± 0.04^a^	0.47 ± 0.01^b^
Total K (TK_s_, g/kg)	7.51 ± 0.33^c^	9.70 ± 0.21^b^	10.84 ± 0.29^a^
Available N (AN_s_, mg/kg)	103.6 ± 31.6^b^	567.3 ± 136.2^a^	560.2 ± 34.9^a^
Available P (AP_s_, mg/kg)	2.31 ± 0.43^b^	13.26 ± 4.93^a^	6.30 ± 1.05^a^
Available K (AK_s_, mg/kg)	55.0 ± 9.1^b^	178.4 ± 48.3^a^	118.8 ± 9.5^a^
Sediment pH (pH_s_)	5.10 ± 0.56^b^	7.15 ± 0.18^a^	5.12 ± 0.41^b^

Data are listed as the mean ± standard deviation (SD). For TK_s_, *n* = 3; for all the other parameters, *n* = 6. Different superscript letters indicate statistically significant differences between sites at the level *P* < 0.05.

### Temperature parameters

**Figure 1:**
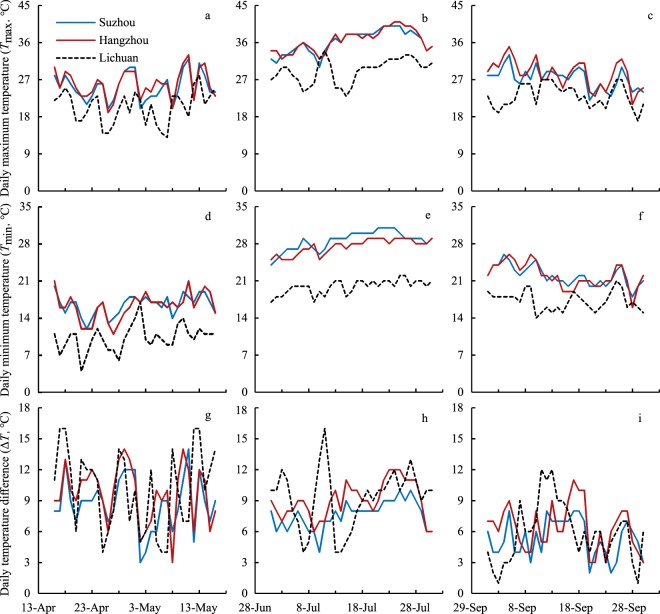
Dynamics of daily maximum temperature (*T*_max_, **a**–**c**), daily minimum temperature (*T*_min_, **d**–**f**) and daily temperature difference (Δ*T*, **g**–**i**) in Suzhou (SZ), Hangzhou (HZ) and Lichuan (LC) across growing seasons.

The dynamic changes in temperature between SZ and HZ during the growing seasons were similar, as they were close to each other in geographical location, whereas those in LC differed significantly (*P* < 0.05) (Fig. 1). The mean values of daily maximum temperature (*T*_max_) and daily minimum temperature (*T*_min_) in SZ and HZ were approximately 5–9 °C higher than those of LC. In summer, the mean values of *T*_max_ were higher than 36 °C in SZ and HZ but was lower than 30 °C in LC. The Daily temperature difference (Δ*T*) in the three locations was approximately 8–10 °C in spring and summer and generally LC > HZ > SZ but was approximately 5–6 °C in autumn and generally HZ > LC > SZ.

### Correlations between plant properties and environmental parameters

The watershield MucC was significantly correlated with the COD_Mn_ and EC_w_ of water (*r* = −0.540 and −0.644, *P* = 0.021 and 0.004, respectively) and the SOC, TN_s_, TK_s_ and AN_s_ of sediments (*r* > 0.585, *P* < 0.05) (Table 4). The MucT was significantly correlated with the COD_Mn_ and TN_w_ of water (*r* = −0.759 and −0.536, *P* < 0.05) and the TK_s_ of sediments (*r* = 0.469, *P* = 0.049). The SBW was significantly correlated with the COD_Mn_ and pH_w_ of water (*r* = −0.598 and 0.523, *P* = 0.009 and 0.026, respectively) but not significantly correlated with sediment parameters (*P* > 0.05). The RLL was significantly correlated with the ORP_w_ and DO_w_ of water (*r* = 0.517 and 0.608, *P* = 0.028 and 0.007, respectively) but not significantly correlated with the sediment parameters (*P* > 0.05). Of the three temperature parameters, both *T*_max_ and *T*_min_ were negatively correlated with the MucC, MucT, SBW and RLL of watershield, but only *T*_min_ was found to have a statistically significant correlation with MucT (*r* = −0.470, *P* = 0.049). Compared with the mucilage traits of MucC and MucT, the plant growth traits SBW and RLL generally showed less connections with the sediments and air temperature. However, except for correlation between MucC and RLL which was not statistically significant, significantly positive correlations were also found among MucC, MucT, SBW and RLL of the plants (data not listed). Significant correlations were also found among parameters of the water and among parameters of the sediments (data not listed).

**Table 4:** Spearman correlation coefficients between mucilage properties and environmental factors (water, sediment and air temperature). Data in bold indicate *P* < 0.05.

Environmental factors		Plant properties			
		MucC	MucT	SBW	RLL
Water	COD_Mn_	**−0.540**	**−0.759**	**−0.598**	−0.218
	TN_w_	−0.342	**−0.536**	−0.457	−0.376
	TP_w_	0.457	0.022	0.013	−0.275
	pH_w_	0.023	0.137	**0.523**	0.137
	ORP_w_	0.170	0.168	0.427	**0.517**
	DO_w_	−0.173	0.071	0.063	**0.608**
	Turb_w_	0.075	0.205	0.030	−0.255
	EC_w_	**−0.644**	−0.271	−0.313	0.030
Sediment	SOC	**0.658**	0.080	0.258	−0.259
	TN_s_	**0.610**	0.066	0.294	−0.149
	TP_s_	0.227	−0.226	−0.071	−0.391
	TK_s_	**0.811**	**0.469**	0.369	−0.075
	AN_s_	**0.586**	0.131	0.259	−0.162
	AP_s_	0.276	−0.124	−0.019	−0.223
	AK_s_	0.362	−0.118	0.068	−0.294
	pH_s_	−0.059	−0.278	−0.146	−0.090
Air temperature	*T*_max_	−0.408	−0.463	−0.383	−0.055
	*T*_min_	−0.406	**−0.470**	−0.432	−0.073
	Δ*T*	0.022	0.019	−0.038	0.014

MucC, mucilage content; MucT, mucilage thickness; SBW, single bud weight; RLL, rolled-leaf length; COD_Mn_, water permanganate index; TN_w_, water total N; TP_w_, water total P; pH_w_, water pH; ORP_w_, water oxidation-reduction potential; DO_w,_ water dissolved oxygen; Turb_w_, water turbidity; EC_w_, water electrical conductivity; SOC, sediment organic carbon; TN_s_, sediment total N; TP_s_, sediment total P; TK_s_, sediment total K; AN_s_, sediment available N; AP_s_, sediment available P; AK_s_, sediment available K; pH_s_, sediment pH; *T*_max_, daily maximum temperature; *T*_min_, daily minimum temperature; Δ*T*, daily temperature difference.

**Figure 2:**
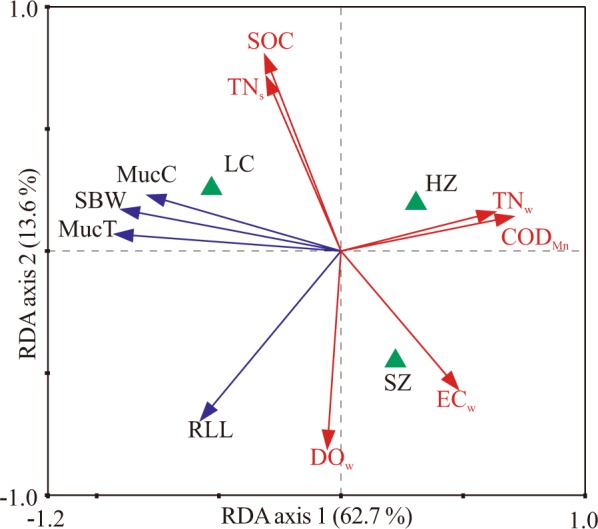
Ordination biplot of redundancy analysis (RDA) displaying the effects of the selected environmental variables on mucilage accumulation. MucC, mucilage content; MucT, mucilage thickness; SBW, single bud weight; RLL, rolled-leaf length; COD_Mn_, water permanganate index; TN_w_, water total N; EC_w_, water electrical conductivity; DO_w_, water dissolved oxygen; SOC, sediment organic carbon; TN_s_, sediment total N; SZ, Suzhou; HZ, Hangzhou; LC, Lichuan.

Redundancy analysis (RDA) can independently retain the contribution of each environmental variable on mucilage accumulation, and it provides another way to estimate the correlation between variables. Based on results from the Monte Carlo permutation test, only the COD_Mn_, TN_w_, EC_w_ and DO_w_ of water and the SOC and TN_s_ of sediments had significant effects on mucilage changes and were selected for the RDA (Fig. 2). The first axis, which explained 62.7% of the variation in mucilage accumulation, was primarily associated with the COD_Mn_, TN_w_ and EC_w_. The second axis, which described 13.6% of the variation in mucilage accumulation, was primarily associated with SOC, TN_s_ and DO_w_. In total, MucC, SBW and MucT increased gradually with decreasing COD_Mn_, TN_w_, EC_w_ and had a slight positive correlation with SOC and TN_s_ but did not show strong relationships with DO_w_. While RLL was positively correlated with DO_w_, it was negatively associated with the rest of the selected environmental variables except EC_w_. LC was characterized by high levels of mucilage accumulation (MucC, SBW, MucT), high SOC and TN_s_, but low COD_Mn_, TN_w_, EC_w_ and DO_w_. HZ had high SOC, TN_s_, COD_Mn_ and TN_w_ but low DO_w_ and EC_w_. SZ showed the opposite condition compared with LC. Both SZ and HZ exhibited similar MucC, SBW and MucT, and the RLL of HZ was lower than that of SZ and LC (Fig. 2).

The RDA results were in accordance with the Spearman correlation analysis. The cumulative percentage variance of plant properties explained by the first four axes of the RDA was 82.2%. The significance of both the first canonical axis and the sum of all canonical axes based on Monte Carlo permutation tests was 0.002, which indicated that the selected environmental variables well explained the mucilage variation, and the remained environmental variables had relatively low explanatory power.

## Discussion

*Brasenia schreberi* is widely cultivated in small ponds and shallow lakes as an aquatic economic crop in East Asia, where young leaves and buds are used as vegetables. The wild distribution of *B*. *schreberi* communities in China is disappearing due to anthropogenic impacts and habitat fragmentation; these communities are ultimately replaced by small cultivation populations, characterized by simple species composition and low biodiversity^25^. The growth and distribution of aquatic macrophytes integrates the chemical, biological and spatiotemporal characteristics of their surrounding environments^31^. In our study, the measured environmental parameters (water, sediments) significantly influenced mucilage accumulation according to RDA and Spearman correlation analysis. Water quality played an important role in mucilage accumulation, followed by sediment nutrient availability. Both MucC and MucT showed negative correlations with COD_Mn_, TN_w_, and EC_w_ but positive correlations with sediment nutrient levels and SOC, which verified our first hypothesis that mucilage accumulation requires good water quality and sediments with high nutrient availability. The temperature parameters showed no significant effect on mucilage accumulation in the RDA, but *T*_max_ and *T*_min_ showed a negative correlation with MucT and SBW in the Spearman correlation analysis, indicating that there may be an optimum temperature range for the mucilage accumulation of *B*. *schreberi* instead of simply negative or positive effects. Excessively high air temperature may limit the growth of *B*. *schreberi*, thus limiting mucilage accumulation.

Among all the tested water quality parameters, COD_Mn_ was the main contributor to mucilage accumulation and showed a significantly negative relationship with MucC and MucT. The influence of COD_Mn_ is likely related to the increase in microorganisms and herbivores with an increase in organic matter in water bodies^32,33^, as mass deaths of *B*. *schreberi* leaves appeared due to dense canopies of the plants. The mucilage that coats the surface of the buds may be consumed by adhered microorganisms, and secondarily, the possible decrease in photosynthetic products due to severe herbivory may result in a decrease in secreted mucilage^30^. Studies showed that increased N loading in water reduced macrophyte biodiversity and changed the structure, and there was a strong effect of N dosing of water on periphyton growth associated with submerged macrophytes at moderately high TP_w_ concentrations^7,34^. Thus, a higher concentration of TN_w_ may favour the growth of periphyton but inhibit the growth of accompanying submerged plants and indirectly affect water quality, which in turn has a negative effect on *B*. *schreberi* growth. Mucilage accumulation was negatively influenced by EC_w_, indicating that mucilage accumulation requires clean water with a low level of salts, as EC_w_ is the reflection of a variety of salts in the water, and a high salt level negatively influenced macrophytes^35,36^. Studies have shown that land use significantly influenced water quality and, as a result, influenced the growth of macrophytes^37–39^. The EC_w_ of LC was significantly lower than that of SZ and HZ, possibly because the irrigation water was from mountain springs purified by the surrounding woodlands. In contrast, the irrigation water in the other two sites was from lakes or reservoirs, which may contain higher salt levels. Extremely low levels of both ORP_w_ and DO_w_ may have a specific negative impact on the growth of *B*. *schreberi* according to the study of Zaman *et al*.^40^, which showed that a variety of metabolic products of *Elodea nuttallii* were significantly negatively affected under hypoxic and anoxic conditions. With our field experience, extremely low ORP_w_ and DO_w_ values in HZ in summer might be the result of dense canopies and mass deaths of leaves on the water surface due to severe self-shading among leaves and high temperature, which was in accordance with the results of Frodge *et al*.^41^. Similarly, for LC in autumn, the decay of leaves in the recession period of growth and the lack of management contributed to the sharp declines in ORP_w_ and DO_w_, which were accompanied by fish deaths. Studies have shown that high Turb_w_ can limit the growth of macrophytes^9,10^; the relationship between Turb_w_ levels and mucilage accumulation in our study was weak, possibly due to the range of Turb_w_ being too small to reflect its effect on the growth of *B*. *schreberi*.

Furthermore, mucilage accumulation showed positive relationships with SOC and sediment nutrient levels in our study. Earlier studies have documented positive relationships between macrophyte growth and sediment organic matter and nutrient availability^4,10,42,43^. In our study, the sites with higher organic carbon and nutrient availability also had higher mucilage content in spring, which may be the result of photosynthesis improvement favoured by higher fertility of sediments. The positive correlations between SOC and all measured nutrients indicated that SOC and sediment nutrients possibly had the same sources.

Global warming changes the physical and chemical characteristics of lakes and catchments^44,45^, and inland surface waters are immediately affected by warming because of the strong correlation between air and surface water temperatures^46^. In other words, variations in water temperature may closely follow the air temperature^47,48^. The daily temperature records from local weather monitoring stations offered us a good opportunity to examine the relationship between watershield quality and air temperature. Although no temperature parameters showed a statistically significant effect on mucilage accumulation according to the RDA results, negative impacts of *T*_max_ and *T*_min_ on the SBW and MucT of *B*. *schreberi* were found based on Spearman analysis, suggesting the possible inhibition of plant photosynthesis or partial solubility of mucilage (for which contains hot water-soluble polysaccharides)^28^, with the average *T*_max_ in both SZ and HZ exceeding 36 °C in summer. In addition, during our field investigation, we found dormant buds, similar to overwintering buds, formed with high air temperature in summer in the cultivation of *B*. *schreberi*. Hence, we suggest that global climate warming may have negative effects on the growth of *B*. *schreberi* and may change its distribution. To reverse the diminishing *B*. *schreberi* populations, strategies are needed to reduce the impacts of anthropogenic activities on water and sediment qualities in cultivation areas. Moreover, long-term monitoring is essential to evaluate the potential effects that climate change could have on *B*. *schreberi* populations.

## Conclusion

Three *B*. *schreberi* cultivation sites in China were used to illustrate the relationships between mucilage accumulation and environmental factors (water, sediment and air temperature). COD_Mn_, TN_w_, EC_w_ and SOC were found to be the main factors influencing the mucilage of *B*. *schreberi*, indicating that the mucilage accumulation and growth of *B*. *schreberi* were sensitive to environmental changes. Good water quality and nutrient-enriched sediments favour the mucilage accumulation of *B*. *schreberi*, whereas high air temperature in summer may have a specific negative effect on the growth of *B*. *schreberi*. According to our findings, *B*. *schreberi* populations may face severe degeneration with worldwide eutrophication in wetlands, with increased N loading (TN_w_) in water caused by N run-off from agricultural lands, excessive fishery, and pollutants from urbanization areas, etc. Meanwhile. Global warming may also threaten the survival of the species. For the moment, habitat conservation is indispensable for preventing the endangered *B*. *schreberi* from extinction.

## Methods

### Study sites

**Figure 3:**
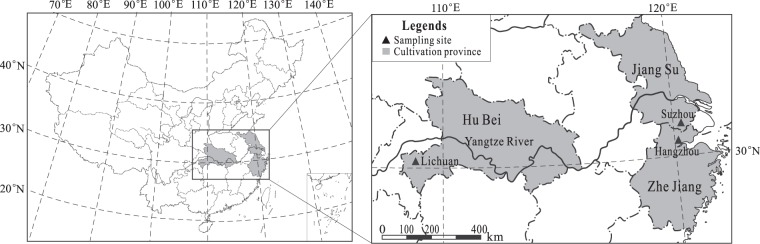
Cultivation locations and sampling sites of *Brasenia schreberi* in China.

Our Sampling sites, SZ and HZ, are located in Jiangsu Province (31°02′N, 120°24′E) and Zhejiang Province (30°11′N, 120°03′E), respectively, in east China (Fig. 3). They are both at an average elevation of 10 m, with a subtropical humid monsoon climate, a frost-free period of 230 (SZ) and 245 (HZ) days and a mean annual precipitation of 1100 (SZ) and 1500 mm (HZ). SZ has an annual mean air temperature of 15.7 °C (minimum 2.5 °C in January and maximum 28.2 °C in July) and HZ has an annual mean air temperature of 17.8 °C (minimum 4.0 °C in January and maximum 28.5 °C in July). LC is located in Hubei Province (30°07′N, 108°49′E) in central China, at an average elevation of 1154 m, with a subtropical continental monsoon climate. It has an annual mean temperature of 12.3 °C (minimum 3 °C in January and maximum 23.5 °C in July), a frost-free period of 232 days and a mean annual precipitation of 1400 mm. To facilitate farming, the watershield cultivation areas are divided into small fields of approximately 500–2000 m^2^ in size. Our sampling sites were selected in those central fields, with sizes of approximately 1000 m^2^ each.

### Field sampling and laboratory analysis

*Brasenia schreberi* and water samples were collected in spring (April 24^th^–May 16^th^), summer (July 14^th^–July 26^th^), and autumn (September 10^th^–September 24^th^) during 2017. Sediment samples were taken during the same time period in spring. Air temperature data were collected to cover a whole month of each sampling season, as April 16^th^–May 16^th^ in spring, July 1^st^–July 31^st^ in summer and September 1^st^–September 30^th^ in autumn.

Four biological properties of *B*. *schreberi*, mucilage content (MucC), mucilage thickness (MucT), single bud weight (SBW) and rolled-leaf length (RLL), were analysed on site, and buds with just one rolled leaf were used. After sampling, SBW, MucT and RLL were immediately measured by a portable electronic scale (OHAUS, SE602FZH, USA) and a Vernier caliper (HENGLIANG, 0–150 mm, China). To determine MucC, the buds were soaked in 0.1 mol/L NaOH for 90 min, and then biomass before and after dissolution was weighed^27^. MucC was calculated as MucC = (biomass_before_−biomass_after_)/biomass_before_ × 100%.

Sampling, preservation, transportation and analysis of the water and sediment samples were performed following standard methods published by the State Environmental Protection Administration of China 2002 (GB3808–2002) and 2004 (HJ/T 166–2004). Given that physical-chemical properties and nutrient constituents are the most important factors influencing water quality, eight water parameters, including electrical conductivity (EC_w_), dissolved oxygen (DO_w_), turbidity (Turb_w_), pH (pH_w_), oxidation-reduction potential (ORP_w_), permanganate index (COD_Mn_), total nitrogen (TN_w_) and total phosphorus (TP_w_) were measured. The first five parameters were analysed on site in the field, as ORP_w_ and pH_w_ using a pH/ORP metre (HANNA, HI98160, Italy), DO_w_ using a dissolved oxygen metre (LEICI, JPBJ-608, China), EC_w_ using a conductivity metre (LEICI, DDB-303A, China) and Turb_w_ using a turbidity metre (XINRUI, WGZ-200B, China). Water samples were taken back to the laboratory for analysis of COD_Mn_, TN_w_ and TP_w_. COD_Mn_ was measured through permanganate oxidation, TN_w_ was determined using the method of persulfate digestion and oxidation through a spectrophotometer (HACH, DR2800, USA) and TP_w_ was analysed through digestion and a colorimetric method (UNICO, UV-2800A, USA).

Eight sediment parameters, organic carbon (SOC), total N (TN_s_), total P (TP_s_), total potassium (TK_s_), available N (AN_s_), available P (AP_s_), available K (AK_s_) and pH (pH_s_), were analysed. Based on Bao^49^, SOC was measured using a potassium dichromate oxidation spectrophotometric method; TN_s_ was measured with the Modified Kjeldahl method and AN_s_ with alkaline hydrolysed diffusion method; TP_s_ was analysed using a Mo-Sb anti spectrophotometric method after wet digestion with H_2_SO_4_ and HClO_4_ and AP_s_ using the adapted-Olsen method; TK_s_ and AK_s_ were analysed using flame photometry after alkali fusion and ammonium acetate extraction, respectively. For pH_s_, 10 g dry sediment was soaked in 25 mL 0.01 mol/L CaCl_2_ solution for 90 min, then the extract was collected to determine pH_s_ using a pH metre (METTLER TOLEDO, FE20, Switzerland).

Air temperature data, including daily maximum and minimum temperature (*T*_max_ and *T*_min_, respectively), were collected from local weather monitoring stations. Then, the daily temperature difference (Δ*T*) was calculated as the difference between *T*_max_ and *T*_min_.

### Data analysis

One-way ANOVA with the Duncan pairwise comparison test was used to compare the seasonal variations or differences between sampling sites in mucilage, water and soil parameters, and Tamhane’s T2 pairwise comparison method was used instead of the Duncan pairwise comparison with heterogeneity of variance. Differences in mucilage, water and sediments affected by sampling sites, seasons and their interactions were examined by multivariate analysis of variance. Spearman correlation analysis and redundancy analysis (RDA) were used to uncover the associations between mucilage properties and various environmental factors (water, sediment and air temperature), with MucC and ORP_w_ data log transformed in the analyses. The statistical analyses mentioned above, with the exception of RDA, were conducted with SPSS 22.0.

RDA with mucilage parameters as response variables and environmental parameters (water, sediment and air temperature) as explanatory variables was applied with the Canoco 4.5 package for Windows, as preliminary detrended correspondence analysis (DCA) indicated that RDA was appropriate for the analysis of mucilage data due to the gradient lengths of <4.0 standard deviations^50^. Response variables were centred and standardized to a zero mean in the analyses. Only explanatory variables showing significance were included in RDA based on Monte Carlo permutation tests (499 permutations) with an alpha level of 0.05, which was also used to examine the significance of the first and all canonical axes.

## Data Availability

The datasets are available from the corresponding author on reasonable request.

## References

[1] Ten Brink P. *et al*. The economics of ecosystems and biodiversity for water and wetlands. Executive Summary. Tech. Rep., London: IEEP (2013).

[2] Mitsch WJ (2013). Wetlands, carbon, andclimate change. Landscape Ecol..

[3] Dhote S, Dixit S (2009). Water quality improvement through macrophytes—a review. Environ. Monit. Assess..

[4] Ogdahl ME, Steinman AD (2014). Factors influencing macrophyte growth and recovery following shoreline restoration activity. Aquat. Bot..

[5] Kissoon LTT (2013). Macrophytes in shallow lakes: Relationships with water, sediment and watershed characteristics. Aquat. Bot..

[6] Akasaka M, Takamura N, Mitsuhashi H, Kadono P (2010). Effects of land use on aquatic macrophyte diversity and water quality of ponds. Freshwater Biol..

[7] Moss B, Jeppesen E, Søndergaard M, Lauridsen TL, Liu Z (2013). Nitrogen, macrophytes, shallow lakes and nutrient limitation: resolution of a current controversy?. Hydrobiologia.

[8] Li K (2017). Exploring the spatial-seasonal dynamics of water quality, submerged aquatic plants and their influencing factors in different areas of a lake. Water.

[9] Rooney RC, Bayley SE (2011). Relative influence of local- and landscape-level habitat quality on aquatic plant diversity in shallow open-water wetlands in Alberta’s boreal zone: direct and indirect effects. Landscape Ecol..

[10] Verhofstad MJJM (2017). Mass development of monospecific submerged macrophyte vegetation after the restoration of shallow lakes: Roles of light, sediment nutrient levels, and propagule density. Aquat. Bot..

[11] Wu Z, Yu D, Wang Z, Li X, Xu X (2015). Great influence of geographic isolation on the genetic differentiation of *Myriophyllum spicatum* under a steep environmental gradient. Sci. Rep..

[12] Kosten S (2009). Climate-related differences in the dominance of submerged macrophytes in shallow lakes. Global Change Biol..

[13] Jeppesen E, Kronvang B, Olesen JE, Audet J, Søndergaard M (2011). Climate change effects on nitrogen loading from cultivated catchments in Europe: implications for nitrogen retention, ecological state of lakes and adaptation. Hydrobiologia.

[14] Silveira MJ, Thiébaut G (2017). Impact of climate warming on plant growth varied according to the season. Limnologica.

[15] Yu D (1991). The geo-historical distribution and ecological adaptation of *Brasenia schreberi* Gmel. In North China. Bull. Bot. Res..

[16] Elakovich SD, Wooten JW (1987). An examination of the phytotoxicity of the water shield. Brasenia schreberi. J. Chem. Ecol..

[17] Kim C, Na HR, Choi H-K (2008). Conservation genetics of endangered *Brasenia schreberi* based on RAPD and AFLP makers. J. Plant Biol..

[18] Dong X (2018). Community structure of B*rasenia schreberi* in the Lichuan reserve in China. Biotic Resour..

[19] Li S, Ke W, Zhu H (2013). Effect of different treatments on seed germination of water shield (*Brasenia schreberi* J. F. Gmel). China Veget..

[20] Lu Z, Zhu J, Zhu S, Chen Z (1984). Preliminary studies on the beetle (*Galerucella birmanica* Jacoby) —an insect pest of waterchestnut and watershield. Sci. Agric. Sin..

[21] Li J, Liu Y, Luo J, Liu P, Zhang C (2012). Excellent lubricating behavior of *Brasenia schreberi* mucilage. Langmuir.

[22] Zhang G, Gao B (2008). Analysis on genetic diversity and genetic structure of *Brasenia schreber*i in Jiangsu and Zhejiang Provinces revealed by ISSRMarkers. J. Lake Sci..

[23] Liu CG (2012). RAPD analysis of genetic diversity and phylogenetic relationships of *Brasenia schreberi* in three producing areas of China. J. Southwest Univ. (Nat. Sci. Ed.).

[24] Kim C (2012). Population genetic structure of the endangered *Brasenia schreberi* in South Korea based on nuclear ribosomal spacer and chloroplast DNA sequences. J. Plant Biol..

[25] Gao B, Zhang G, Chen H (2007). Species diversity of *Brasenia schreberi* community in different habitats. Chin. J. Appl. Ecol..

[26] Wu H, Lv Z, Zhang Z, Yu J (2017). A comparative study of the commercially valuable components of water shield *(Brasenia schreber*i) from 4 cultivation areas in China. J. Southwest Univ. (Nat. Sci. Ed.).

[27] Kakuta M, Misaki A (1979). Polysaccharide of “Junsai (*Brasenia schreberi* J. F. Gmel)” mucilage: Constitution and linkage analysis. Agric. Biol. Chem..

[28] Zhou Y, Wu Y, Tang Q, Zhou Z (2005). Separation of vitro gmel and primary analysis of its composition in water shield. Food Fermentation Ind..

[29] Liu P (2014). Mechanism of biological liquid superlubricity of *Brasenia schreberi* mucilage. Langmuir.

[30] Thompson KA, Sora DM, St. Cross KS, Germain JM, Cottenie K (2014). Mucilage reduces leaf herbivory in Schreber’s watershield, *Brasenia schreberi* J.F. Gmel. (Cabombaceae). Botany.

[31] Alahuhta J (2015). Geographic patterns of lake macrophyte communities and species richness at regional scale. J. Veg. Sci..

[32] Rösel S, Rychła A, Wurzbacher C, Grossart H-P (2012). Effects of pollen leaching and microbial degradation on organic carbon and nutrient availability in lake water. Aquat. Sci..

[33] Zhang Y (2015). Water organic pollution and eutrophication influence soil microbial processes, increasing soil respiration of estuarine wetlands: site study in Jiuduansha wetland. Plos One.

[34] Özkan K, Jeppesen E, Johansson LS, Beklioglu M (2010). The response of periphyton and submerged macrophytes to nitrogen and phosphorus loading in shallow warm lakes: a mesocosm experiment. Freshwater Biol..

[35] Roache MC, Bailey PC, Boon PI (2006). Effects of salinity on the decay of the freshwater macrophyte. Triglochin procerum. Aquat. Bot..

[36] James KR, Hart BT, Bailey PCE, Blinn DW (2009). Impact of secondary salinisation on freshwater ecosystems: effect of experimentally increased salinity on an intermittent floodplain wetland. Mar. Freshwater Res..

[37] Lougheed VL, Crosbie B, Chow-Fraser P (2001). Primary determinants of macrophyte community structure in 62 marshes across the Great Lakes basin: latitude, land use, and water quality effects. Can. J. Fish. Aquat. Sci..

[38] Feijoó CS, Lombardo RJ (2007). Baseline water quality and macrophyte assemblages in Pampean streams: A regional approach. Water Res..

[39] Cañedo-Argüelles M (2016). Saving freshwater from salts. Science.

[40] Zaman T, Asaeda T (2013). Effects of NH_4_-N concentrations and gradient redox level on growth and allied biochemical parameters of *Elodea nuttallii* (Planch.). Flora.

[41] Frodge JD, Thomas GL, Pauley GB (1990). Effects of canopy formation by floating and submerged aquatic macrophytes on the water quality of two shallow Pacific Northwest lakes. Aquat. Bot..

[42] Carr GM, Chambers PA (1998). Macrophyte growth and sediment phosphorus and nitrogen in a Canadian prairie river. Freshwater Biol..

[43] Xie D, Yu D, Yu LF, Liu CH (2010). Asexual propagations of introduced exotic macrophytes *Elodea nuttallii*, *Myriophyllum aquaticum*, and *M*. *propinquum* are improved by nutrient-rich sediments in China. Hydrobiologia.

[44] Hamilton DP, Salmaso N, Paerl HW (2016). Mitigating harmful cyanobacterial blooms: strategies for control of nitrogen and phosphorus loads. Aquat. Ecol..

[45] Yankova Y, Neuenschwander S, Köster O, Posch T (2017). Abrupt stop of deep water turnover with lake warming: Drastic consequences for algal primary producers. Sci. Rep..

[46] Dokulil MT (2013). Impact of climate warming on European inland waters. Inland Waters.

[47] O’Reilly, C. M. *et al*. Rapid and highly variable warming of lake surface waters around the globe. *Geophys*. *Res*. *Lett*. **42**, 10.1002/2015GL066235 (2015).

[48] Isaak DJ, Wollrab S, Horan D, Chandler G (2012). Climate change effects on stream and river temperatures across the northwest U.S. from 1980–2009 and implications for salmonid fishes. Climatic Change.

[49] Bao, S. *Soil Agrochemical Analysis*. (China Agricultural Press, Beijing, 2010).

[50] Ter Braak, C. J. F. & Šmilauer, P. *Canoco Reference Manual and CanoDraw for Windows User’s Guide: software for canonical community ordination (version 4*.*5*). (Microcomputer Power, Ithaca, NY, 2002).

